# Amino Acid Assisted Incorporation of Dye Molecules within Calcite Crystals

**DOI:** 10.1002/anie.201804365

**Published:** 2018-06-10

**Authors:** Bartosz Marzec, David C. Green, Mark A. Holden, Alexander S. Coté, Johannes Ihli, Saba Khalid, Alexander Kulak, Daniel Walker, Chiu Tang, Dorothy M. Duffy, Yi‐Yeoun Kim, Fiona C. Meldrum

**Affiliations:** ^1^ School of Chemistry University of Leeds Woodhouse Lane Leeds LS2 9JT UK; ^2^ School of Physics and Astronomy University of Leeds Woodhouse Lane Leeds LS2 9JT UK; ^3^ School of Physics & Astronomy University College London Gower Street London WC1E 6BT UK; ^4^ Diamond Light Source Ltd Harwell Science & Innovation Campus Didcot OX11 0DE UK

**Keywords:** biomimetics, biomineralization, calcium carbonate, crystal growth, nanocomposites

## Abstract

Biomineralisation processes invariably occur in the presence of multiple organic additives, which act in combination to give exceptional control over structures and properties. However, few synthetic studies have investigated the cooperative effects of soluble additives. This work addresses this challenge and focuses on the combined effects of amino acids and coloured dye molecules. The experiments demonstrate that strongly coloured calcite crystals only form in the presence of Brilliant Blue R (BBR) and four of the seventeen soluble amino acids, as compared with almost colourless crystals using the dye alone. The active amino acids are identified as those which themselves effectively occlude in calcite, suggesting a mechanism where they can act as chaperones for individual molecules or even aggregates of dyes molecules. These results provide new insight into crystal–additive interactions and suggest a novel strategy for generating materials with target properties.

With their versatility and ease of application, soluble additives are widely used to control crystallisation processes. There is no better demonstration of the power of this approach than the process of biomineralisation, in which organisms achieve exceptional control over mineral formation to generate structures such as bones and seashells.^1^ While this is achieved by many biogenic strategies, all are united by one common feature: the use of organic molecules.^2^ Significant efforts have therefore been made to identify biomolecules associated with specific roles such as selecting crystal polymorphs and tuning crystal textures, and to translate the underlying principles to synthetic systems.^3^ Many small organic molecules,^4^ and larger species such as polyelectrolytes^5^ and block copolymers4a, 6 have to‐date been identified that are active in controlling crystallisation processes.

While the focus of these studies has principally been on the activities of individual additives, crystals in biology typically grow under the influence of a suite of additives. This has the potential for greater control, where individual additives could be deployed at different time‐points in the reaction,^7^ or they could act in combination to deliver an outcome that could not be achieved with individual additives. While a number of bio‐inspired studies have demonstrated the cooperative effects of soluble additives and insoluble organic matrices,^8^ few have addressed the effects of combinations of soluble additives,^9^ possibly due to the challenge of investigating a large reaction space.

This article describes an investigation into the crystallisation of calcium carbonate in the presence of mixtures of organic additives, where we focus on the combined effects of amino acids and coloured dye molecules. The selection of a coloured dye as one of our additives is crucial to our strategy, where it allows us to go beyond basic properties such as morphology and use optical microscopy to determine how the additives cooperate to drive occlusion within the crystal lattice.^10^ Such occlusion is key to the superior mechanical properties of single‐crystal biominerals,^11^ to the generation of characteristic crystallographic textures,3a, 12 and can be used to generate novel nanocomposites.^13^ Our study shows that certain amino acids can vastly increase the occlusion of a common dye—Brilliant Blue R (BBR)—within calcite, generating strongly coloured single crystals. This demonstrates that selected combinations of additives can facilitate the formation of composite materials that would not be accessible with traditional methods and proposes an incorporation mechanism that is relevant to biological and synthetic systems.

Calcium carbonate was precipitated by mixing equal volumes of 10 mm solutions of CaCl_2_ and NaHCO_3_, and adding aliquots of amino acids and BBR at concentrations that yielded single crystals of calcite. Crystallisation was then allowed to proceed for two days. In all cases the polymorphs generated were confirmed by using powder XRD and Raman microscopy (Figures S1 and S2). The synergistic effects of the amino acids and dyes can be immediately seen at typical reaction conditions of [Ca^2+^]:[amino acid]:[dye]=250:25:1 (Figures 1 a and e). Very weakly coloured rhombohedral calcite crystals precipitated in the presence of BBR alone, while aspartic acid (Asp) alone generated calcite crystals that were elongated along the *c*‐axis, as is characteristic of this additive.^11^, ^14^ In combination, however, these additives generated intensely blue calcite crystals whose morphologies were unchanged as compared with Asp alone. Identical results were obtained using either d‐ or l‐Asp.

**Figure 1:**
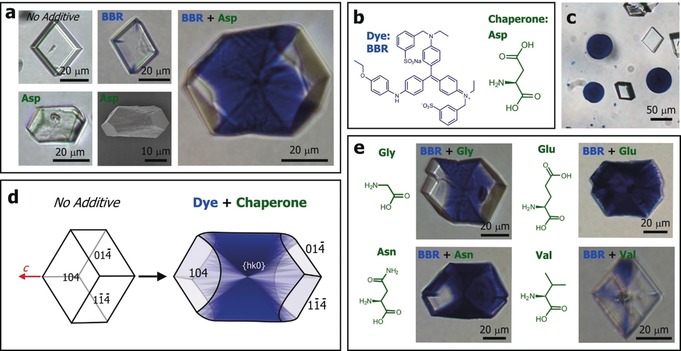
CaCO_3_ crystals precipitated in the presence of Brilliant Blue R (BBR) and amino acids. a) Optical microscope and SEM images of calcite crystals precipitated in the presence of BBR and aspartic acid (Asp), as indicated. b) The structures of BBR and Asp. c) Colourless calcite rhombohedra and coloured vaterite crystals precipitated in the presence of BBR alone. d) Schematic diagrams showing the occlusion of BBR/amino acids within the equatorial zone. e) Calcite crystals grown in the presence of BBR and the amino acids glycine (Gly), glutamic acid (Glu), asparagine (Asn) and valine (Val) under conditions of [Ca^2+^]:[asp]:[dye]=250:25:1 and [Ca^2+^]:[Glu, Gly, Asn, Val]:[dye]=250:50:1.

A small number of vaterite particles were sometimes observed and were always strongly coloured, even in the absence of Asp (Figure 1 c). This is due to the polycrystalline structure of the vaterite, where the dye is entrapped between the crystalline units. Strong colouration of single crystals, in contrast, only occurs if the dye becomes occluded within the crystal lattice. That dye was incorporated within the calcite crystals was confirmed by washing them with sodium hypochlorite to degrade any surface‐bound molecules, dissolving them in acid, and then characterising the resulting solution using UV/Vis spectroscopy. Crystals were also embedded in epoxy resin and polished to expose their interiors, where optical microscopy and energy‐dispersive X‐ray analysis (EDX) confirmed occlusion (Figure S3).

Further examination of the dyed calcite crystals showed that many exhibited zoning effects, where the dye was preferentially associated with the {*hk*0}^15^ faces that define the equatorial zone (Figure 1 d). A transmitted light image therefore appears as an hour‐glass structure. Non‐uniform occlusion within calcite can be attributed to differential adsorption of additives to the acute and obtuse step edges,^16^ where the amino acids that give occlusion here are expected to preferentially bind to the acute over the obtuse steps. This effect will be less prominent at higher concentrations of amino acids when binding to both acute and obtuse steps occurs.^17^ Such anisotropic partitioning of molecules within calcite has previously been observed for small ions such as Mg^2+^,^18^ Sr^2+^ and SO_4_^2−^,^19^ and larger fluorescent dyes.^17^, ^20^

The generality of these cooperative effects was then explored by screening all 17 soluble amino acids with BBR under the same reaction conditions. Only 4 amino acids supported significant BBR occlusion: aspartic acid (Asp), glycine (Gly), glutamic acid (Glu), and asparagine (Asn) (Figures 1 a and e) and all exhibited zoning; that the crystal containing Glu/ BBR appears to be uniformly coloured in Figure 1 is due to the intense colour. For comparison, an image is also shown of a crystal grown in the presence of valine (Val)(Figure 1 e). This amino acid does not occlude in calcite, and supports little dye incorporation. A number of initial experiments were also performed to explore whether this “chaperone” strategy is unique to BBR. Figure 2 shows that Asp is indeed active in driving the occlusion of 3 of 7 other dyes tried (where their structures are shown in Figure S4), demonstrating the potential value of this synthetic approach.

**Figure 2:**
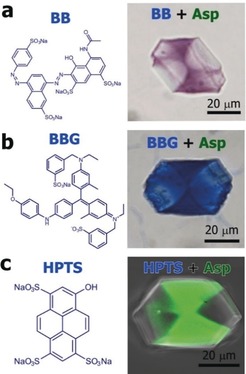
Calcium carbonate crystals precipitated in the presence of Asp and Brilliant Black (BB), Brilliant Blue G (BBG) or 8‐hydroxypyrene‐1,3,6‐trisulfonic acid (HPTS), under conditions of [Ca^2+^]:[asp]:[BB]=250:25:0.5 and [Ca^2+^]:[asp]:[BBG, HPTS]=250:25:1. Non‐uniform occlusion is seen.

Having demonstrated that Asp effectively chaperones BBR into calcite, we investigated the effects on the crystal lattice using synchrotron high‐resolution powder X‐ray diffraction (HR‐PXRD). The data were analysed using Rietveld analysis (Figure 3). Calcium carbonate was precipitated under conditions [Ca^2+^]=5 mm and [Ca^2+^]:[Asp]:[BBR]=250:25:1 and in the presence of Asp or BBR alone. All samples comprised calcite, together with 7–13 % vaterite, and the calcite crystals exhibited coherence lengths comparable to geological calcite (550–700 nm). Calcite crystals precipitated in the presence of Asp alone comprised 0.26 wt % Asp and exhibited a peak shift towards lower angles due to lattice distortions of Δ*c*/*c*=3.05×10^−4^ and Δ*a*/*a*=5.21×10^−5^, whereas calcite containing Asp and BBR displayed smaller lattice distortions of Δ*c*/*c*=1.86×10^−4^ and Δ*a*/*a*=1.40×10^−5^, but broader peaks. This anisotropic lattice distortion is consistent with the elastic anisotropy of calcite.^11^ Calcite crystals precipitated in the presence of BBR alone exhibited a small increase in peak width, which is indicative of low levels of occlusion, while those formed at higher Asp concentrations ([Ca^2+^]:[amino acid]:[dye]=250:250:1) exhibited greater lattice distortions, but no further peak broadening (Figure S5). These data show that the peak shifts were exclusively due to the incorporated Asp, but that BBR contributes to peak broadening. Similar effects have been observed for calcite containing a range of nanoparticles.^21^

**Figure 3:**
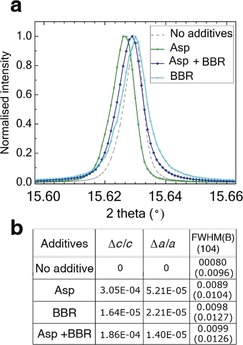
HR‐PXRD patterns of calcite crystals grown at [Ca^2+^]=5 mm and [Ca^2+^]:[Asp]:[BBR]=250:25:1, recorded at *λ*=0.826252 Å. a) The {104} reflection from crystals containing no additives, Asp alone, BBR alone and BBR and Asp. b) Lattice distortions and Full Width Half Maximum (FWHM) and B (internal breadth) induced by the additives with respect to pure calcite.

Further information about the cooperation between Asp and BBR was obtained by using in situ atomic force microscopy (AFM) using a liquid cell (Figure 4), where the methods are described in detail in the Supporting Information. AFM analysis was performed under slow flow of four variants of the calcium carbonate solutions:^22^ an additive‐free solution and solutions containing Asp only, BBR only, and both Asp and BBR. [Ca^2+^]=1.2 mm and the concentrations of Asp and BBR were set at [Ca^2+^]:[amino acid]: [dye]=100:1:1. Calcite grows via a step‐growth mechanism under these conditions, and steps originate from screw dislocations present on the {104} faces (Figure 4 a).

**Figure 4:**
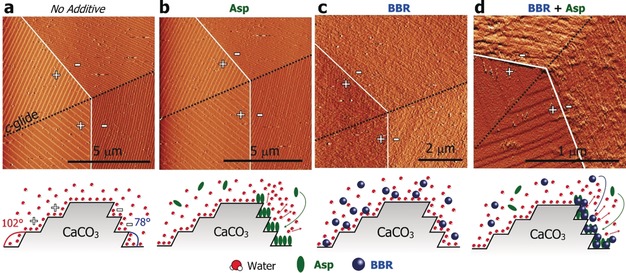
a–d) AFM images of calcite crystals grown in the presence of additives recorded using a liquid cell, and associated schematic images of the additive‐binding that occurs under each condition. a) Screw dislocation observed for a crystal grown in the absence of additives displayed well defined step boundaries (white) and *c*‐glide (black) + and −signs indicate obtuse and acute steps, respectively. b) Little change occurs in the step shape following introduction of low concentrations of Asp to the growth solution, where Asp binds preferentially to acute step edges(see Figure S6). c) when BBR is the only additive, it is strongly adsorbed to the calcite {104} faces and shows no preference for either step edge. d) BBR and Asp act in combination to preferentially bind to the acute steps. a, b) were obtained in contact mode, whereas c, d) were obtained in Tapping Mode.

Addition of Asp to the reaction solution at these concentrations had minor effects on the shapes of the steps and their rates of propagation (Figures 4 b). Preferential binding to the acute steps was seen at higher Asp concentrations, as is consistent with the literature (Figure S6). BBR, in contrast, adsorbed to all exposed faces (Figure 4 c). The images are indicative of the low solubility BBR forming aggregates in solution, as occurs for many dyes. Addition of a mixture of Asp and BBR molecules to the growth solution, in contrast, caused severe roughening of the acute $\left\langle 44\bar{1}\right\rangle$ and $\left\langle 44\bar{8}\right\rangle$ steps, while most of the obtuse steps preserved their well‐defined shapes (Figure 4 d). This pattern of behaviour is also characteristic of Asp alone at higher Asp concentrations, where binding to the acute over the obtuse steps occurs due to an enhanced stereo‐chemical fit.^16^ Asp and BBR therefore together have a greater effect on calcite growth than identical concentrations of the individual additives.

These data demonstrate that certain amino acids can drive the occlusion of BBR in calcite, where minimal occlusion occurs in their absence. But what is the mechanism by which this takes place? Of the 17 amino acids tested, Asp, Glu, Gly and Asn were the most active. Notably, these activities correlate closely with their occlusion efficiencies in calcite.4c, 11, 23 This suggests three potential scenarios, where (1) the active amino acids and BBR may complex in solution, such that the amino acid chaperones BBR into the crystal lattice, (2) the amino acids retard crystal growth, where the longer residence time of strongly binding additives at step edges/kink sites gives greater occlusion and (3) these amino acids facilitate direct binding of BBR to the crystal surface, facilitating occlusion.

The first scenario was investigated using Molecular Dynamics (MD) simulations. These were performed at 300 K and utilised the AMBER force field to model the dye and amino acid molecules.^24^ Asp was assigned a net charge of −1, where this is consistent with the experimental pH of ≈8.5. The calculations showed that there was no association between Asp and BBR, where any transient complexes rapidly separated. Multiple other variables including changes in the pH (where this was achieved by assigning a different charge to the amino acid) and the addition of a Ca^2+^ ion to the Asp molecules were also explored, but no association between BBR and Asp was ever observed. Given that Asp and BBR do not associate in bulk solution—when they have full conformational freedom—we find it extremely unlikely that BBR complexes to Asp molecules bound to the calcite surface. NMR spectroscopy was also explored, but the solubility of BBR in water was too low to provide any information on Asp‐BBR interactions.

We are also able to rule out the second scenario, where no visible increase in BBR occlusion was obtained on reduction of the calcium concentration (and thus supersaturation). In addition, analysis of the quantities of Asp and BBR occluded showed that the amount of BBR occluded correlates with the amount of Asp occluded, rather than the amount of Asp in solution (Figure 5), where the latter would be expected if dye occlusion occurred due to a retardation of the growth rate.

**Figure 5:**
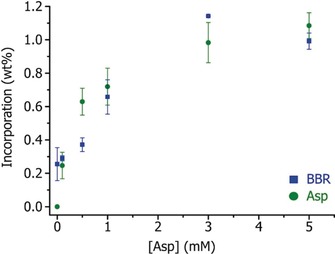
Graph showing the amounts of BBR and Asp occluded within calcite crystals as a function of [Asp] in solution at [BBR]=0.04 mm and [Ca^2+^]=5 mm.

Considering then the third scenario, the binding of amino acids to calcite has previously been studied using simulations and experiments. A complex picture emerges which shows that occlusion efficiency cannot be predicted based on binding strength to the crystal surface. Under the experimental conditions employed here, the amino acids are zwitterionic and preferentially bind through the carbonyl oxygen and the amide hydrogen of the peptide functionality.^16^, ^25^ Alanine, Asp, Glu, Gly, leucine and tyrosine all bind to calcite in water at pH 8 to a significant degree,^26^ and Asp and Gly exhibit almost identical adsorption free energies.25b However, there are enormous differences in their incorporation efficiencies and Asp is occluded far more efficiently than Gly at identical concentrations.^23^

Simulations provide an explanation for these effects. Asp binds directly to the calcite surface, stabilised by interactions of the side‐chain carboxy group with local water molecules.25c Asp may therefore promote BBR occlusion by disrupting the first hydration layer and allowing its intimate association with the crystal surface. Gly, in contrast, is thought to substitute for water molecules in the *second hydration layer* rather than binding directly to the mineral surface—from where it cannot be occluded.^27^ However, that Gly is effectively occluded at high solution concentrations indicates that a proportion of the adsorbed molecules must displace water molecules to bind directly to the calcite surface. As BBR alone is occluded at very low levels suggests that it does not bind directly to the calcite surface. This mechanism is illustrated schematically in Figure 4. Further modelling and experimental work is required to fully understand the complex mechanisms at play.

Finally, it is interesting to consider the generality of this mechanism. That the amino acids can chaperone aggregates of BBR into the crystal lattice likely contributes to the strong colouration observed. We therefore performed additional experiments with Asp and the highly soluble fluorescent dye HPTS (8‐hydroxypyrene‐1,3,6‐trisulfonic acid) to confirm that Asp is equally effective in driving the occlusion of individual molecules in calcite. Confocal fluorescence microscopy of the product crystals confirmed that the dye is uniquely located in the zone occupied by Asp (Figure 2)—that is, that defined by binding to acute steps—which shows that this “partnership” mechanism can potentially be applied to a wide range of additives.

This work demonstrates that greater control over the growth and properties of crystals can be achieved if organic additives are employed in combination, rather than individually. Focusing on the attractive test‐system of a coloured dye (BBR) and amino acids, we show that four amino acids—those that are themselves effectively occluded in calcite—drive the occlusion of BBR at levels that vastly exceed those achieved with the dye alone. Importantly, our work also shows that these cooperative effects are not restricted to the BBR/Asp partnership. These results therefore suggest a novel strategy for generating materials with target properties, where combinatorial methods9b, 28 and high‐throughput screening approaches4c could be employed to rapidly explore the large reaction space created by multiple additives. They are also of particular relevance to biomineralisation processes, where multiple additives are invariably present within the biological environment.

## Conflict of interest

The authors declare no conflict of interest.

## Supporting information

As a service to our authors and readers, this journal provides supporting information supplied by the authors. Such materials are peer reviewed and may be re‐organized for online delivery, but are not copy‐edited or typeset. Technical support issues arising from supporting information (other than missing files) should be addressed to the authors.

Supplementary
