# Supplementary material for: Amino Acid Assisted Incorporation of Dye Molecules within Calcite Crystals
Source: Angew Chem Int Ed Engl. 2018 Jun 10;57(28):8623–8. doi: 10.1002/anie.201804365 (PMC6055892; doi:10.1002/anie.201804365)
Supplement: Supplementary file 1 — Supplementary [file ANIE-57-8623-s001.pdf]

Angewandte Chemie
Eine Zeitschrift der Gesellschaft Deutscher Chemiker

Supporting Information

Amino Acid-Assisted Incorporation of Dye Molecules within Calcite Crystals

*Bartosz Marzec, David C. Green, Mark A. Holden, Alexander S. Coté, Johannes Ihli, Saba Khalid, Alexander Kulak, Daniel Walker, Chiu Tang, Dorothy M. Duffy, Yi-Yeoun Kim, and Fiona C. Meldrum**

anie_201804365_sm_miscellaneous_information.pdf

1. Materials and Methods

1.1 Materials

All chemicals used were purchased from Sigma-Aldrich at the highest available purity grade and were used without further purification. These include calcium chloride dihydrate, sodium bicarbonate, boric acid, potassium hydroxide, L-alanine, glycine (Gly), L-valine (Val), L-tyrosine, L-aspartic acid sodium salt dihydrate (Asp), L-glutamic acid sodium salt dihydrate (Glu), L-arginine, L-histidine, L-lysine, L-serine, L-threonine, L-tryptophan, L-cysteine, L-asparagine (Asn), L-proline, L-methionine, L-glutamine, ethylenediamine tetraacetic acid disodium dihydrate (EDTA), isobutryl-l-cysteine (IBLC), *o*-phthaldialdehyde (OPA), Brilliant Blue R sodium salt (BBR), Brilliant Blue G sodium salt (BBG), Brilliant Black BN tetra-sodium salt (BB), Brilliant Violet 5R trisodium salt, Fast Green FCF, 8-hydroxypyrene-1, 3, 6-trisulfonic acid tri sodium salt (HPTS), Brilliant Yellow 3GP, and Brilliant Green. Deionised (DI) water was obtained from an in-house Millipore Reference A+ water purification system (MilliQ, 1-2 ppm OC, 18.2 mΩ).

1.2 Experimental Methods

Precipitation of Calcite Crystals. Initially, aqueous stock solutions of $CaCl_2$ (10 mM), $NaHCO_3$ (10 mM), all amino acids examined (50 mM) and all dyes examined (2 mM) were prepared in 250 mL volumetric flasks with DI water and stored in glass bottles. A Ca^{2+}/amino acid/dye solution was prepared in a 15 mL glass vial charged with a glass substrate (sections of glass microscope slides cleaned with Piranha solution, and rinsed with DI water before drying under a flow of air). This was prepared by mixing 5 mL of 10 mM solutions of $CaCl_2$ with aliquots of amino acids and dyes added as required. Once prepared, 5 mL 10 mM $NaHCO_3$ stock solution was added to initiate the reaction and precipitate $CaCO_3$ for 2 days at room temperature.

When the amino acid Asp was used, the ratio between [Ca^{2+}]:[Asp]:[dye] was maintained at 250:25:1, such that [Ca^{2+}] = 5 mM, [Asp] = 0.5 mM, and [dye] = 0.02 mM. This was achieved with the addition of 100 µL 50 mM amino acid stock and 100 µL 2 mM dye stock. This ratio was applied for colouration with dyes Brilliant Blue R (BBR), Brilliant Blue G (BBG), 8-hydroxypyrene-1, 3, 6-trisulfonic acid (HPTS), Fast Green FCF, Brilliant Violet 5R trisodium salt, Brilliant Green and Brilliant Yellow 3GP (BY). For Brilliant Black BN (BB), the dye concentration was halved, and the ratio 500:50:1 ([Ca^{2+}] = 5 mM, [Asp] = 0.5 mM, and [dye] = 0.01 mM) was used instead. For other amino acids (including Glu, Asn, Gly and Val; amongst others), the amino acid concentration was doubled such that [Ca^{2+}]:[amino acid]:[dye] was set at 250:50:1, such that [Ca^{2+}] = 5 mM, [amino acid] = 1.0 mM, and [dye] = 0.02 mM. Volumes of stock solution added were adjusted accordingly. The vial was then covered with a plastic lid

punched several times with a needle and left for 2 days. After that time the glass substrate was removed from the vial, washed with a 14% solution of sodium hypochlorite for 1 h, water, then ethanol, and then dried in a stream of air.

Large-scale Preparation of Calcite Crystals: In order to generate sufficient material for pXRD and dye/amino acid quantification analysis, calcite crystals were grown in 5 L beakers for 7 days in a reaction between $CaCl_2$ and $NaHCO_3$, where the final concentrations of both reagents were 5 mM. Amino acids and dyes were added as required, at the same [Ca^{2+}]:[amino acid]:[dye] ratios as used before. In an example experiment, a 2 L aqueous solution containing 10 mM $CaCl_2$, 1 mM Asp and 0.04 mM BBR was prepared, before a final 2 L 10 mM $NaHCO_3$ solution was added to yield 4 L of crystallisation liquor. Crystals were collected from the base of the beakers with a Pasteur pipette, centrifuged and washed several times with a 14% solution of sodium hypochlorite to remove surface-bound organics, rinsed with DI water, sonicated in ethanol for 2 h, followed by a final rinse in ethanol. For experiments involving a fixed BBR concentration and changing Asp concentration, final concentrations [BBR] = 0.04 mM, [Ca^{2+}] = 5 mM and [Asp] = 0 mM, 0.5 mM, 1.0 mM, 3.0 mM or 5.0 mM were used.

1.3 Molecular Dynamics Simulations

Molecular Dynamics simulations were employed to identify any potential structural fragments of Asp and BBR that would bind the molecules together. Simulations were carried out at 300 K for up to 0.5 ns, with a timestep of 0.5 fs, using DL_POLY_4.[2] The AMBER force field, which has well-defined bond lengths, bond angles, partial charges and van der Waals parameters, was used for all of the amino acids and dye molecules[3] and the flexible SPC model was used for water.[4] The Asp was relaxed in the vicinity of the frozen BBR molecule, starting from a number of favourable initial configurations. Water was then added to the most favorable associated configurations and allowed to equilibrate for 100 ps at 300 K, while keeping the dye molecule and amino acid frozen. All constraints were then relaxed and the simulation was run for 0.5 ns at 300 K.

1.4 Analytical Methods

The sizes and morphologies of the precipitated crystals were determined using optical microscopy and scanning electron microscopy (SEM). Optical microscopy images were recorded using a Nikon Eclipse LV100 microscope equipped with transmitted and reflected light sources. In a typical procedure, glass substrates supporting crystals were covered with a thin layer of UV-transparent and fluorescence free immersion oil to increase the image quality. SEM was carried out using an FEI Nova 450 NanoSEM microscope equipped with a CBS detector. The glass substrates were mounted on aluminium stubs

using copper tape, and were imaged uncoated. Imaging was performed using low landing voltage (0.5 – 2 kV) and high deceleration voltage (2-4 kV). Energy-Dispersive X-Ray (EDX) mapping was performed using TEAM EDS Analysis system for SEM. In order to collect EDX maps, the beam accelerating voltage was increased to 15 kV and the beam deceleration was turned off. Maps were collected for 30 minutes using medium resolution analysis (256 x 256 pixels).

Modelling of crystal morphologies for generating graphical descriptions of approximate faces was conducted on WinXMorph.[5]

Identification of the distribution of fluorescent dyes (i.e. HPTS) in calcite was performed with confocal fluorescence microscopy. Confocal fluorescence microscopy was conducted using a Zeiss LSM510 Upright Confocal Microscope using samples grown directly on clean glass substrates in under oil immersion where required. Lasers (488 nm) and filters (505 nm low pass filter) were selected based on their suitability to the excitation and emission maxima of HPTS. Image rendering and analysis was conducted in ImageJ.

Identification of crystal polymorph was carried out using Raman microscopy and powder XRD (PXRD). Raman spectra were recorded using a Renishaw Raman Microscope equipped with a 785 nm laser, while laboratory PXRD experiments were carried out using a PANalytical X'Pert^3 diffractometer equipped with a Cu-anode (λ = 1.54056 Å). Samples were deposited on Si substrates and data were collected at ambient conditions in the 2θ range between 5° and 50°. The recorded datasets were analysed using HighScore Plus software suite.

High resolution synchrotron PXRD experiments were performed at beam line I11 at Diamond Synchrotron Radiation Facility (Diamond Light Source Ltd, Didcot, UK). Instrument calibration were performed with silicon standards and samples were loaded into borosilicate glass capillaries (Φ = 0.5 mm) and mounted on a spinner. The beam wavelength was 0.825969 Å (15 keV) and data were recorded using the Multiple Array of Crystals (MAC) detector in the 2Θ range between 5° and 150° with 0.001° step size. Lattice parameters and structural parameters were refined by the Rietveld refinement method $\approx$20 peaks in the 2Θ range between 5° and 40° to obtain quantitative information about the polymorphs, lattice parameters and distortion, coherence lengths and strains. The FWHM and internal breadth of major reflections were also analysed by line profile analysis using Topas Academic and Panalytical X'Pert HighScore Plus software.

Dye content of coloured crystals was measured by quantifying the concentration of dye from dissolved crystals using UV-Vis spectroscopy, and comparing the mass of dye in solution to the mass of crystals

dissolved**.** UV-Vis spectra were recorded using a Thermo Scientific Nanodrop 2000c. Reference solutions of concentrations 0 mM, 0.01 mM, 0.05 mM, 0.1 mM and 0.5 mM BBR were prepared in an aqueous solution containing 200 mM EDTA disodium salt and 0.66 M potassium borate buffer (pH 10.4). The absorbances at 465 nm (background), 550 nm, 560 nm, 570 nm and 595 nm, of each solution were then recorded, and used to prepare a calibration curve (A vs [BBR]). Known masses of coloured calcite crystals containing BBR were then dissolved in 5 mL of an aqueous solution containing 200 mM EDTA disodium salt and 0.66 M potassium borate buffer (pH 10.4) and analysed in the same way for the reference solutions. Measured absorbances were compared to the calibration curve to determine BBR concentration, which in turn is used to determine the extent of incorporation in wt%.

Amino acid content of coloured crystals was measured by quantifying the concentration of amino acids from dissolved crystals using fluorescence spectroscopy, and comparing the mass of amino acid in solution to the mass of crystals dissolved. A previously published fluorescence-based assay was adapted to provide accurate measurements of the extent of amino acid incorporation in calcite single crystals. We measured the mass of crystals before dissolution in aqueous EDTA solution to release the incorporated amino acid into solution. The amino acid was derivitised to yield a fluorescent reporter. The fluorescence intensity of each well was then measured and contrasted against a calibration curve of known amino acid concentration to determine the number of moles of amino acid molecules, and therefore mass of amino acid, in a given volume. This value was compared against the known mass of $CaCO_3$ to provide an extent of incorporation given in wt%.

For analysis, a 96-well plate (Greiner µClear Black) was charged with 100 µL of known concentrations (0, 0.02, 0.05, 0.1, 0.2, 0.3, 0.4, 0.5 mM in a buffer containing 200 mM EDTA disodium salt and 0.66 M potassium borate (pH 10.4)) for each amino acid as reference samples; and 100 µL of each dissolved crystal solution as prepared for dye quantification was pipetted into separate wells. Then each reference well and sample well is treated with 100 µL derivatisation solution (0.1 g isobutyryl-L-cysteine (IBLC) and 0.045 g o-phthaldialdehyde (OPA) in 20 mL 1 M pH 10.4 potassium borate buffer (26 mM IBLC and 17 mM OPA)) and analysed immediately.

***Liquid cell Atomic Force Microscope (AFM) Experiments*.** AFM images were recorded using a Bruker Multimode 8 with a NanoScope V controller. Images were collected using silicon nitride cantilevers with nominal spring constants of 0.35 $N \cdot m^{-1}$ (Bruker SNL-10). Rhombohedral calcite seed crystals ≈ 80 µm in size were pre-precipitated onto glass cover slips under additive-free conditions by mixing equal volumes of 10 mM $CaCl_2$ and $NaHCO_3$ aqueous solutions. *In situ* measurements were collected whilst flowing supersaturated calcium carbonate growth solutions over the seed crystals at 0.1 - 0.25 mL min^{-1}. The influence of the additives on the calcite growth was investigated by growing the calcite

seeds in the presence and absence of additives, using a fixed calcium concentration and ionic strength, as used previously by Cho *et al*.[1] The growth solutions provided stable, slow growth conditions which can be effectively studied on an AFM time-scale. The mole ratio between Ca^{2+} ions, L-asp and BBR was kept constant at 250:1:1. *In situ* imaging was performed in contact mode or Tapping Mode™ where appropriate.

References

[1] K.-R. Cho, Y.-Y. Kim, P. Yang, W. Cai, H. Pan, A. N. Kulak, J. L. Lau, P. Kulshreshtha, S. P. Armes, F. C. Meldrum, J. J. De Yoreo, *Nat. Commun.* **2016**, *7*, 10187.

[2] I. T. Todorov, W. Smith, K. Trachenko, M. T. Dove, *J. Mater. Chem.* **2006**, *16*, 1911-1918.

[3] W. D. Cornell, P. Cieplak, C. I. Bayly, I. R. Gould, K. M. Merz, D. M. Ferguson, D. C. Spellmeyer, T. Fox, J. W. Caldwell, P. A. Kollman, *J. Am. Chem. Soc.* **1995**, *117*, 5179-5197.

[4] Y. Wu, H. L. Tepper, G. A. Voth, *J. Chem. Phys.* **2006**, *124*, 0245031.

[5] aW. Kaminsky, *J Appl Crystallogr* **2005**, *38*, 566-567; bW. Kaminsky, *J. Appl. Crystal.* **2007**, *40*, 382-385.

2. Supplementary Figures

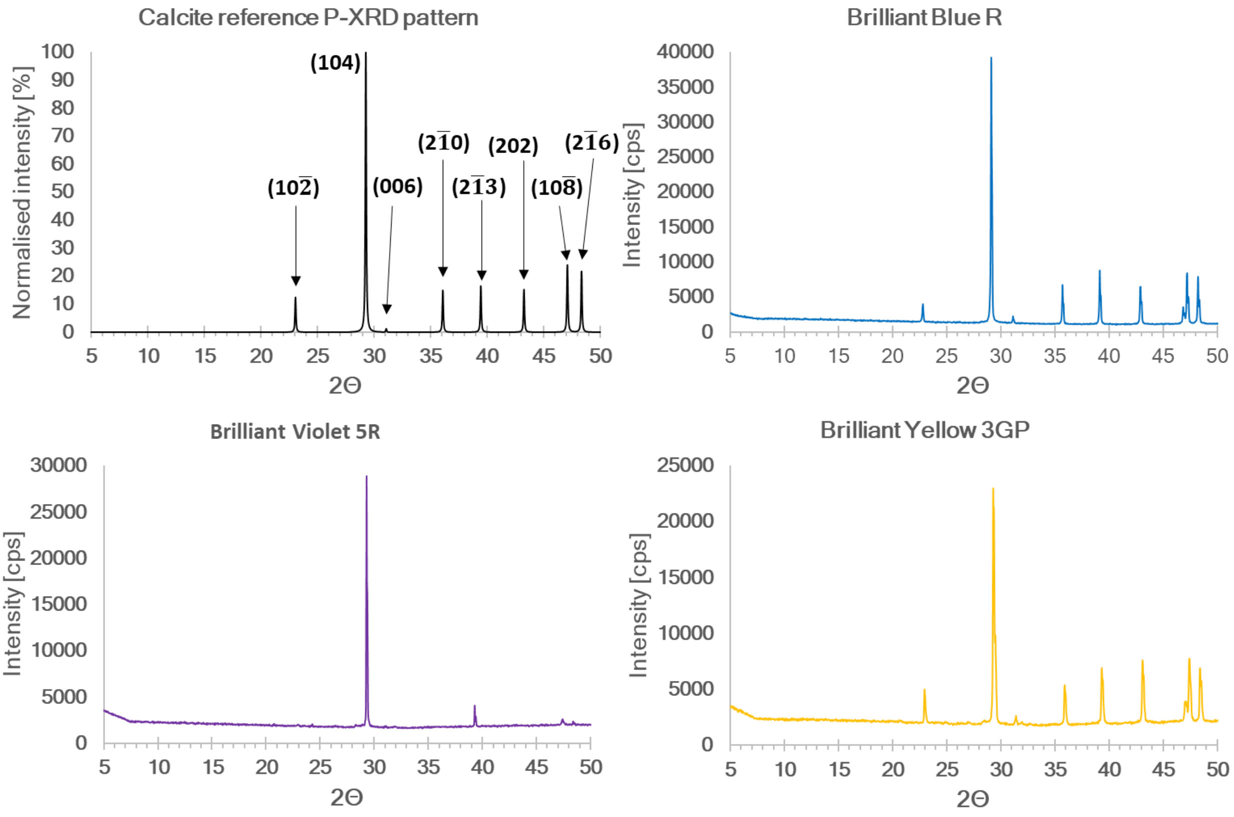

Figure S1. PXRD patterns of additive-free and coloured calcite crystals. The pattern from the additive-free crystals was simulated and indexed using synchrotron single crystal X-ray diffraction data obtained from the American Mineralogist Crystal Structure Database.

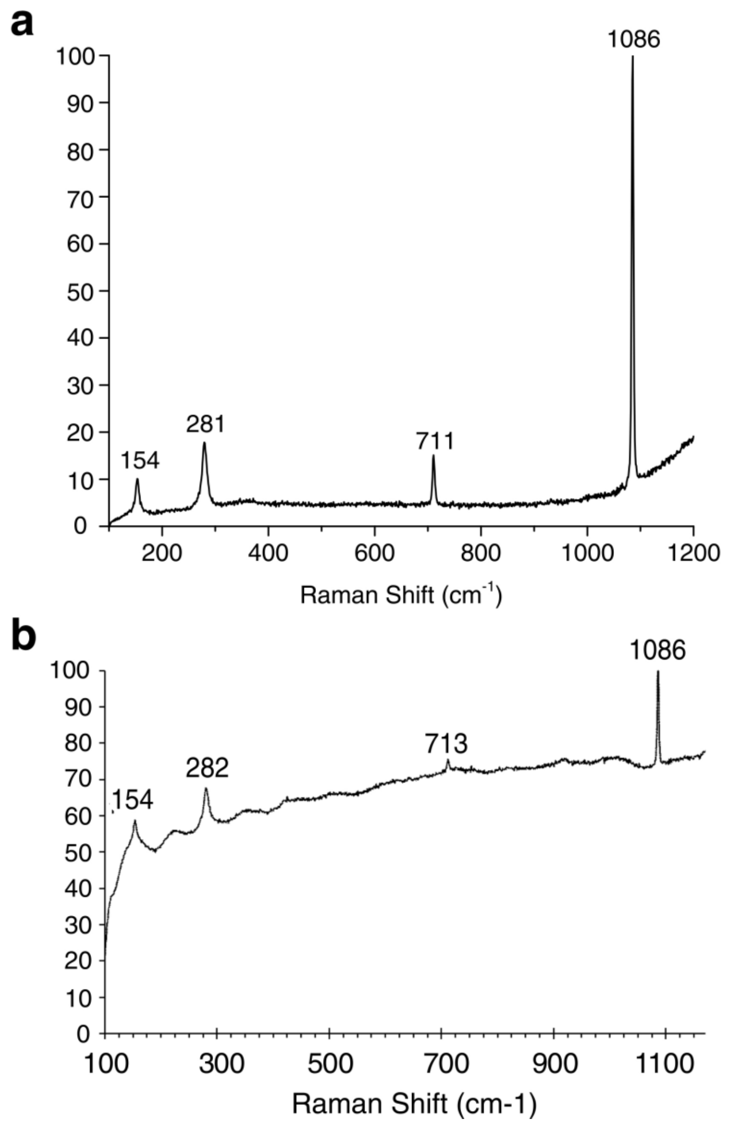

Figure S2. Raman spectra of (a) additive-free and (b) coloured calcite crystals. Both the control and coloured crystals were demonstrated to be calcite with the presence of peaks at 154 cm^{-1} (lattice mode), 282 cm^{-1} (lattice mode), 713 cm^{-1} (bending) and 1086 cm^{-1} (stretching). (b) The high florescence background in the spectrum from the coloured calcite crystals is due to the occluded organic dyes.

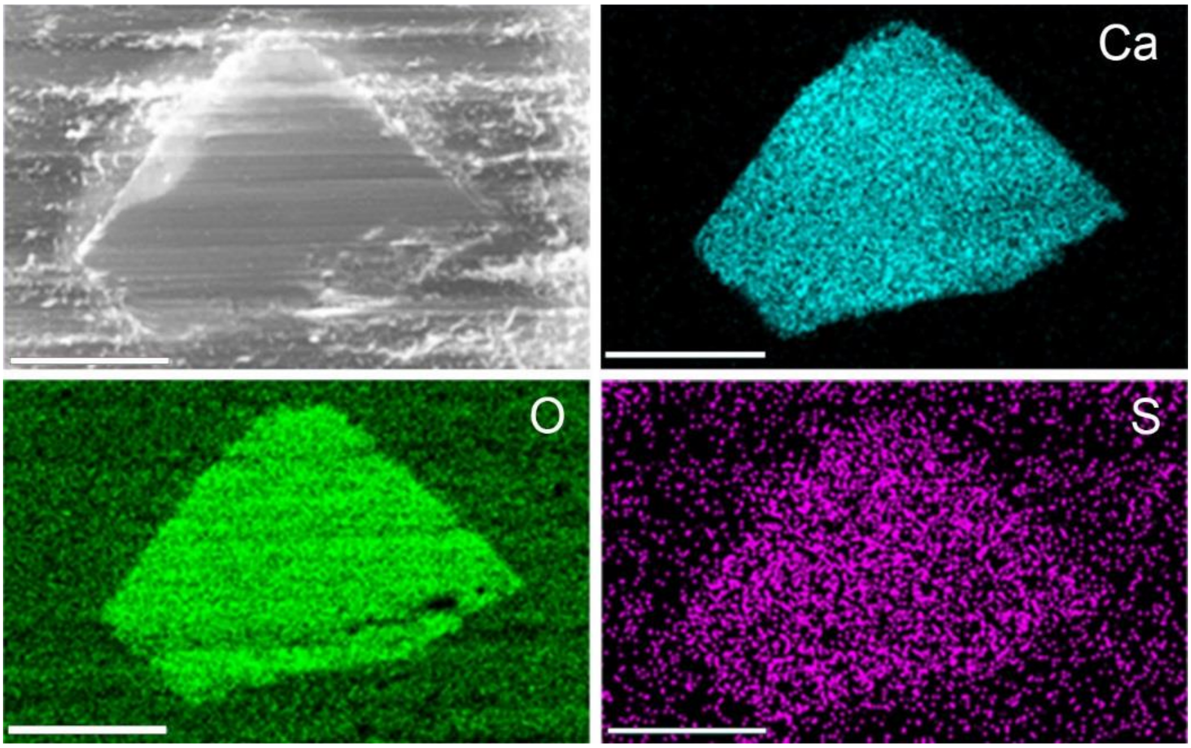

Figure S3. EDX mapping of a coloured calcite crystal precipitated in the presence of Asp and BBR. Analysis was performed of uncoated crystals, embedded within epoxy resin and polished to reveal the interior, which shows a uniform distribution of dye, as indicated by the sulphur signal. Scale bar: 20 µm.

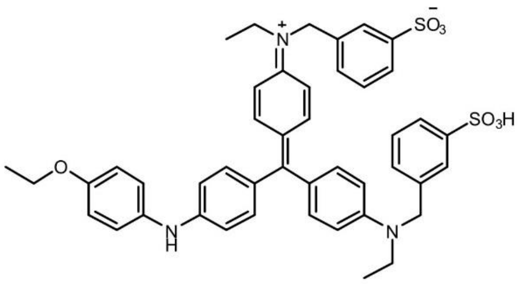

Brilliant Blue R (BBR)

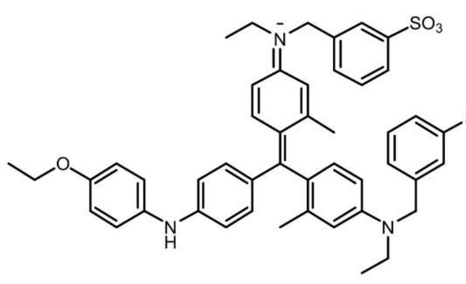

Brilliant Blue G (BBG)

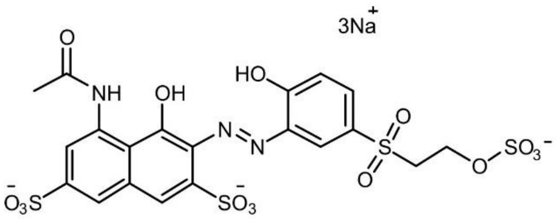

Brilliant Violet 5R

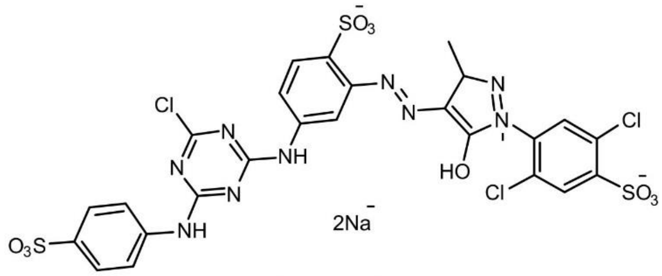

Brilliant Yellow 3GP

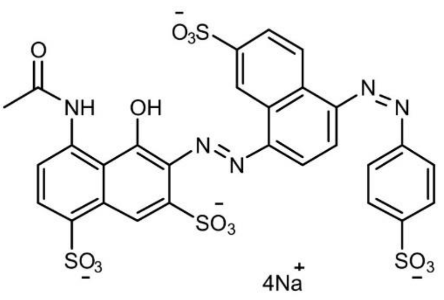

Brilliant Black BN

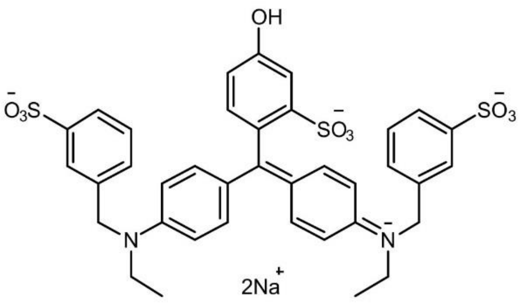

Fast Green FCF

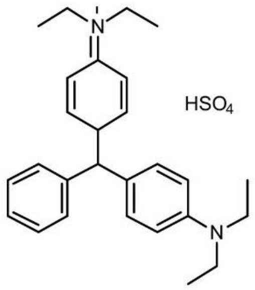

Brilliant Green

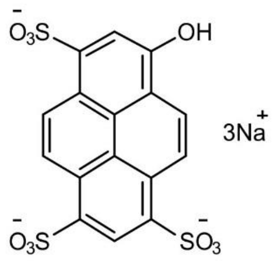

8-Hydroxypyrene-1,3,6-trisulfonic acid (HPTS)

Figure S4. Structures of all of the different dyes explored in combination with Asp for occlusion, where incorporation was only observed for BBR, BB, BBG and HPTS. It is noted that HPTS is fluorescent and can therefore be detected at significantly lower levels of occlusion than the coloured dyes.

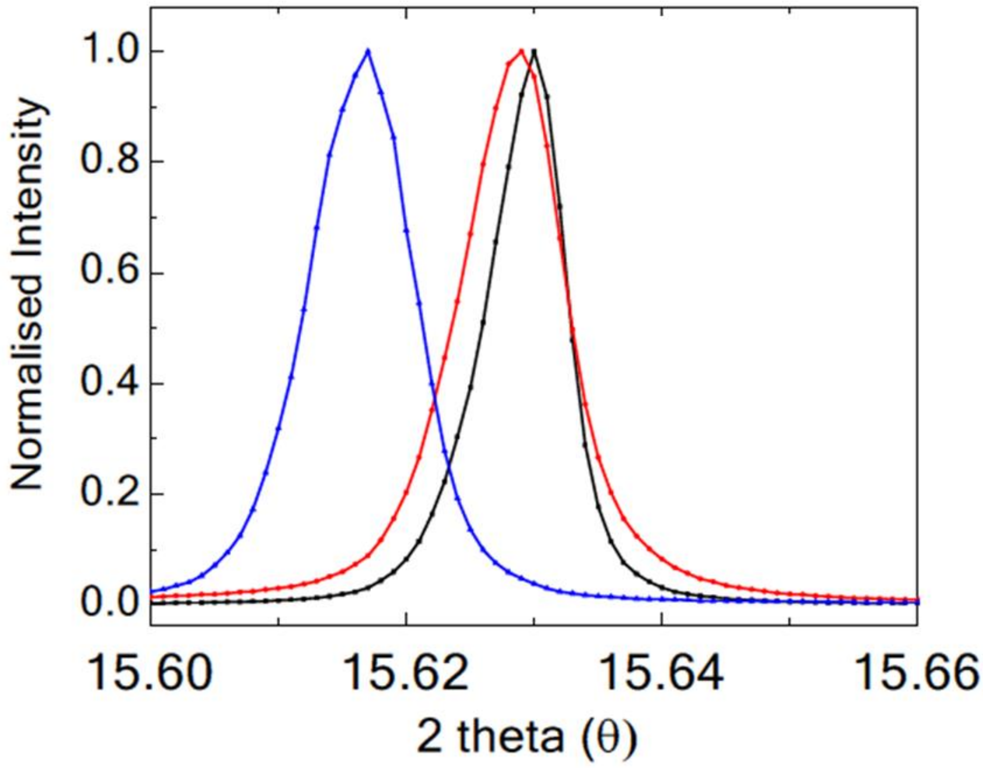

Figure S5. The {104} XRD reflections of calcite crystals containing BBR and Asp obtained from HR-XRD measurements. Crystals were precipitated from solutions of composition [Ca^{2+}] = 5 mM with no additives (pure calcite, black), [Ca^{2+}] = 5 mM and [Ca^{2+}]:[Asp]:[BBR] = 250:25:1 (red) and [Ca^{2+}] = 20 mM and [Ca^{2+}]:[Asp]:[BBR] = 250:250:1 (blue). The observed peak shifts towards lower angles is due to increased incorporation of Asp.

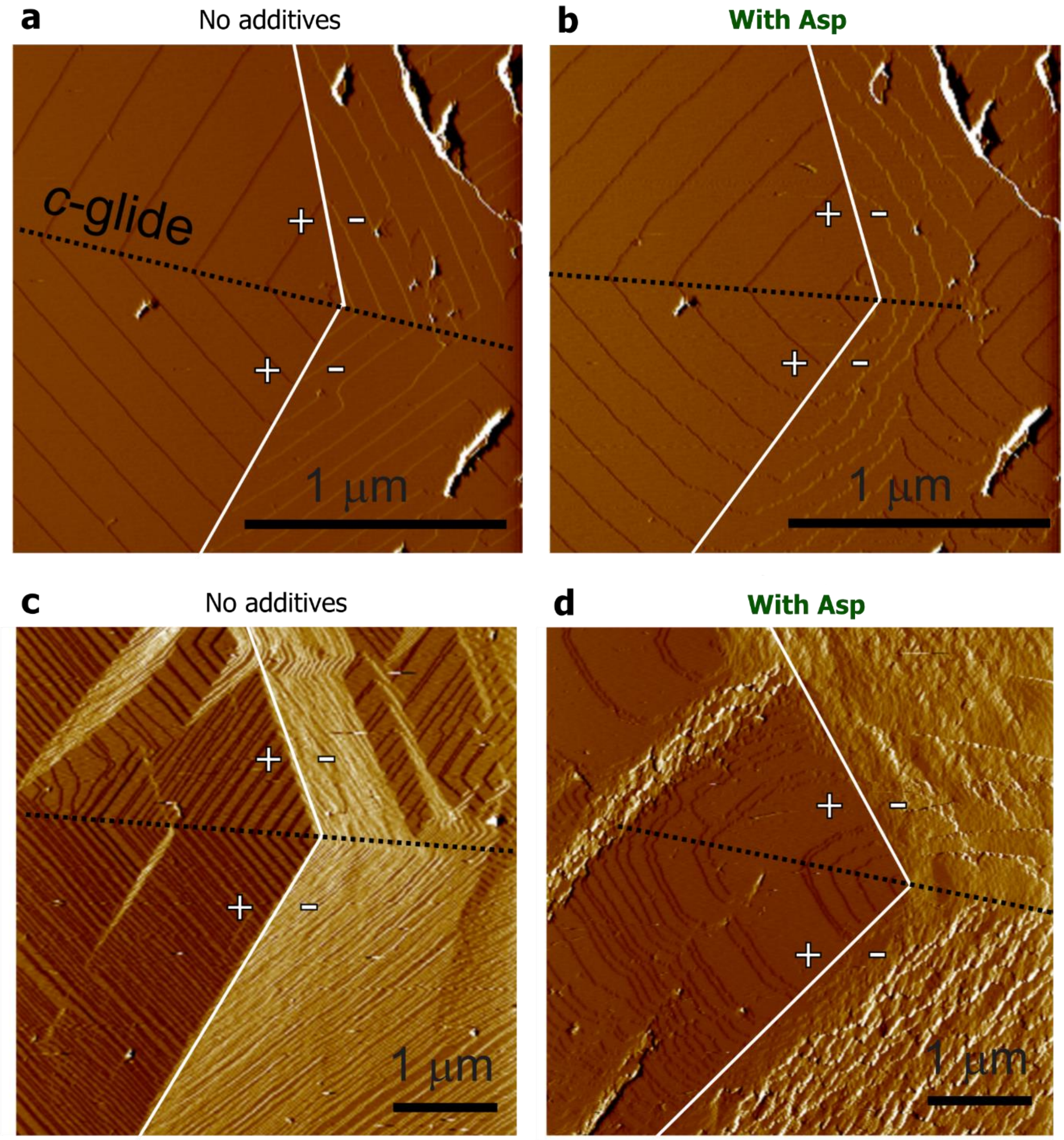

Figure S6: (a-d) *In situ* AFM contact mode micrographs of calcite crystals growing in the absence and presence of Asp. (a) Screw dislocation observed for a crystal grown in the absence of additives displayed well defined step boundaries and c-glide. (b) At [Asp]:[Ca] = 1:1, significant alteration of the acute steps occurs due to the binding of Asp. (c) Screw dislocation observed for a crystal grown in the absence of additives at a higher supersaturation than in (a). (d) Addition of Asp significantly alters the acute step morphology, causing step rounding and bunching.
